# *N* ^2^-*o*-Tolyl­benzamidine

**DOI:** 10.1107/S1600536808017625

**Published:** 2008-06-19

**Authors:** Li-Zeng Zhang, Hong-Bo Tong

**Affiliations:** a Institute of Applied Chemistry, Shanxi University, Shanxi 030006, People’s Republic of China

## Abstract

The asymmetric unit of the title compound, C_14_H_14_N_2_, contains two independent mol­ecules with slightly different conformations; the dihedral angles formed by aromatic rings in the two mol­ecules are 73.2 (1) and 75.0 (1)°. Inter­molecular N—H⋯N hydrogen bonds link the mol­ecules into chains extended in the [100] direction.

## Related literature

For general background, see Bourget-Merle *et al.* (2002 ▶). For a related crystal structure, see Surma *et al.* (1988 ▶).
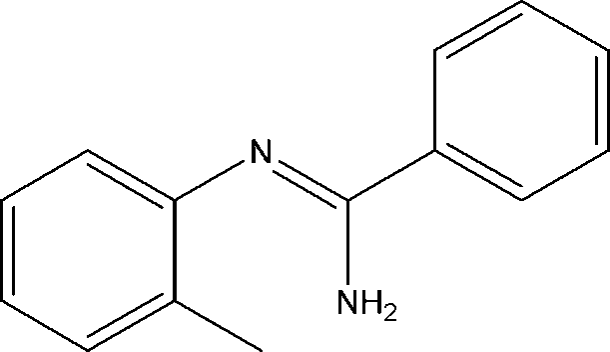


## Experimental

### 

#### Crystal data

C_14_H_14_N_2_
*M* *_r_* = 210.27
Triclinic, 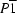

*a* = 10.347 (2) Å
*b* = 10.697 (2) Å
*c* = 11.495 (2) Å
α = 97.088 (4)°
β = 103.184 (4)°
γ = 95.898 (4)°
*V* = 1218.0 (4) Å^3^
*Z* = 4
Mo *K*α radiation
μ = 0.07 mm^−1^
*T* = 298 (2) K
0.30 × 0.20 × 0.20 mm

#### Data collection

Siemens SMART CCD area-detector diffractometer
Absorption correction: multi-scan (*SADABS*; Sheldrick, 1997 ▶) *T*_min_ = 0.980, *T*_max_ = 0.986
4978 measured reflections
4158 independent reflections
2913 reflections with *I* > 2σ(*I*)
*R*_int_ = 0.021

#### Refinement

*R*[*F* ^2^ > 2σ(*F* ^2^)] = 0.073
*wR*(*F* ^2^) = 0.235
*S* = 1.09
4158 reflections
291 parameters
H-atom parameters constrained
Δρ_max_ = 0.44 e Å^−3^
Δρ_min_ = −0.46 e Å^−3^

### 

Data collection: *SMART* (Siemens, 1996 ▶); cell refinement: *SAINT* (Siemens, 1996 ▶); data reduction: *SAINT*; program(s) used to solve structure: *SHELXS97* (Sheldrick, 2008 ▶); program(s) used to refine structure: *SHELXL97* (Sheldrick, 2008 ▶); molecular graphics: *ORTEP-3* (Farrugia, 1997 ▶); software used to prepare material for publication: *SHELXL97*.

## Supplementary Material

Crystal structure: contains datablocks I, global. DOI: 10.1107/S1600536808017625/cv2419sup1.cif

Structure factors: contains datablocks I. DOI: 10.1107/S1600536808017625/cv2419Isup2.hkl

Additional supplementary materials:  crystallographic information; 3D view; checkCIF report

## Figures and Tables

**Table 1:** Hydrogen-bond geometry (Å, °)

*D*—H⋯*A*	*D*—H	H⋯*A*	*D*⋯*A*	*D*—H⋯*A*
N2—H2*B*⋯N3	0.86	2.25	3.049 (3)	156
N4—H4*B*⋯N1^i^	0.86	2.24	3.016 (3)	151

Symmetry code: (i) 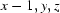.

**Fig. 1.**
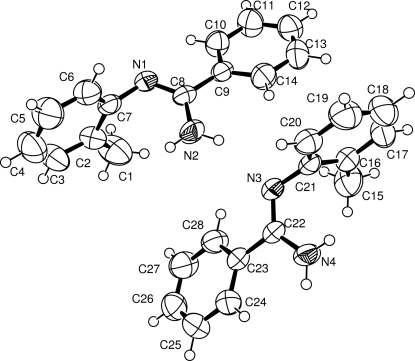
Two independent molecules of (I) showing the atom-labelling scheme. Displacement ellipsoids are drawn at the 50% probability level.

## supplementary crystallographic information

### Comment

β-Diketiminate complexes are among the most common chelate systems in coordination chemistry (Bourget-Merle *et al.*, 2002). Inspired by getting new chelate system with amidine motif, we got the title compound (I) by additional reaction of PhCN with *o*-methyl aniline lithium.

The asymmetric unit of (I) contains two independent molecules (Fig. 1), denoted A and B. The N=C bond lengths in both molecules (Table 1) agree well with the corresponding values reported for similar compounds (Surma *et al.*, 1988). The conformations of the two independent molecules are slightly different. In molecule A, the mean plane N3/C22/N4 makes dihedral angles of 85.3 (1) and 21.5 (1)° with phenyl rings C16–C21and C23–C28, respectively. In molecule B, the mean plane N1/C8/N2 makes dihedral angles 86.8 (1) and 18.2 (1)° with phenyl rings C2–C7 and C9–C14, respectively.

In the crystal, intermolecular N—H···N hydrogen bonds (Table 1) link the molecules into chains extended in direction [100].

### Experimental

All experiments were performed under an atmosphere of pure argon using Schlenk apparatus and a vacuum line, unless otherwise stated. The solvents used were of reagent grade or better and were freshly distilled under dry dinitrogen and degassed prior to use. Slowly added PhCN(1.03 g,10 mmol)to the solution of compound *o*-methyl-PhNHLi (1.13 g,10 mmol) in hexane (*ca* 40 ml)at -0°C., and then stirred for further 12 h.Add it to cold water, and then use chlorform to extract organic phase.The organic phase was slowly concentrated and get the crystal of the title compound.

### Refinement

The H atoms were positioned geometrically and allowed to ride on their parent atoms, with N—H = 0.86 Å, C—H = 0.93–0.97 Å, and *U*_iso_ = 1.2–1.5 *U*_eq_(parent atom).

### Crystal data

**Table:**

C_14_H_14_N_2_	*Z* = 4
*M**_r_* = 210.27	*F*_000_ = 448
Triclinic, *P*1	*D*_x_ = 1.147 Mg m^−^^3^
Hall symbol: -P 1	Mo *K*α radiation λ = 0.71073 Å
*a* = 10.347 (2) Å	Cell parameters from 2024 reflections
*b* = 10.697 (2) Å	θ = 2.4–27.7º
*c* = 11.495 (2) Å	µ = 0.07 mm^−^^1^
α = 97.088 (4)º	*T* = 298 (2) K
β = 103.184 (4)º	Plate, colourless
γ = 95.898 (4)º	0.30 × 0.20 × 0.20 mm
*V* = 1218.0 (4) Å^3^	

### Data collection

**Table:**

Siemens SMART CCD area-detector diffractometer	4158 independent reflections
Radiation source: fine-focus sealed tube	2913 reflections with *I* > 2σ(*I*)
Monochromator: graphite	*R*_int_ = 0.021
*T* = 298(2) K	θ_max_ = 25.0º
ω scans	θ_min_ = 1.8º
Absorption correction: multi-scan(SADABS; Sheldrick, 1997)	*h* = −10→12
*T*_min_ = 0.980, *T*_max_ = 0.986	*k* = −12→12
4978 measured reflections	*l* = −13→13

### Refinement

**Table:**

Refinement on *F*^2^	Secondary atom site location: difference Fourier map
Least-squares matrix: full	Hydrogen site location: inferred from neighbouring sites
*R*[*F*^2^ > 2σ(*F*^2^)] = 0.073	H-atom parameters constrained
*wR*(*F*^2^) = 0.235	*w* = 1/[σ^2^(*F*_o_^2^) + (0.1497*P*)^2^] where *P* = (*F*_o_^2^ + 2*F*_c_^2^)/3
*S* = 1.09	(Δ/σ)_max_ < 0.001
4158 reflections	Δρ_max_ = 0.44 e Å^−^^3^
291 parameters	Δρ_min_ = −0.46 e Å^−^^3^
Primary atom site location: structure-invariant direct methods	Extinction correction: none

### Special details

**Table:**

Geometry. All e.s.d.'s (except the e.s.d. in the dihedral angle between two l.s. planes) are estimated using the full covariance matrix. The cell e.s.d.'s are taken into account individually in the estimation of e.s.d.'s in distances, angles and torsion angles; correlations between e.s.d.'s in cell parameters are only used when they are defined by crystal symmetry. An approximate (isotropic) treatment of cell e.s.d.'s is used for estimating e.s.d.'s involving l.s. planes.
Refinement. Refinement of *F*^2^ against ALL reflections. The weighted *R*-factor *wR* and goodness of fit *S* are based on *F*^2^, conventional *R*-factors *R* are based on *F*, with *F* set to zero for negative *F*^2^. The threshold expression of *F*^2^ > σ(*F*^2^) is used only for calculating *R*-factors(gt) *etc*. and is not relevant to the choice of reflections for refinement. *R*-factors based on *F*^2^ are statistically about twice as large as those based on *F*, and *R*- factors based on ALL data will be even larger.

### Fractional atomic coordinates and isotropic or equivalent isotropic displacement parameters (Å^2^)

**Table:**

	*x*	*y*	*z*	*U*_iso_*/*U*_eq_	
N1	0.90491 (17)	0.79986 (19)	0.88806 (17)	0.0538 (5)	
N2	0.69224 (19)	0.8197 (2)	0.9148 (2)	0.0688 (6)	
H2A	0.6726	0.8582	0.8530	0.083*	
H2B	0.6343	0.8055	0.9564	0.083*	
C1	0.7749 (4)	0.6552 (3)	0.6545 (3)	0.1043 (12)	
H1A	0.7196	0.6274	0.5744	0.156*	
H1B	0.7251	0.6362	0.7126	0.156*	
H1C	0.8527	0.6121	0.6659	0.156*	
C2	0.8172 (3)	0.7949 (3)	0.6711 (2)	0.0644 (7)	
C3	0.8023 (3)	0.8623 (3)	0.5739 (3)	0.0852 (9)	
H3	0.7654	0.8180	0.4965	0.102*	
C4	0.8387 (4)	0.9873 (4)	0.5865 (3)	0.0972 (11)	
H4	0.8248	1.0285	0.5189	0.117*	
C5	0.8971 (4)	1.0561 (3)	0.7000 (3)	0.0927 (10)	
H5	0.9236	1.1433	0.7096	0.111*	
C6	0.9149 (3)	0.9920 (3)	0.7983 (3)	0.0680 (7)	
H6	0.9543	1.0371	0.8749	0.082*	
C7	0.8762 (2)	0.8639 (2)	0.7862 (2)	0.0508 (6)	
C8	0.81298 (19)	0.7817 (2)	0.9464 (2)	0.0478 (5)	
C9	0.8415 (2)	0.7144 (2)	1.05346 (19)	0.0490 (6)	
C10	0.9449 (3)	0.6419 (2)	1.0709 (2)	0.0647 (7)	
H10	0.9974	0.6353	1.0152	0.078*	
C11	0.9718 (3)	0.5790 (3)	1.1693 (3)	0.0786 (8)	
H11	1.0427	0.5313	1.1796	0.094*	
C12	0.8962 (3)	0.5858 (3)	1.2516 (3)	0.0787 (8)	
H12	0.9148	0.5427	1.3177	0.094*	
C13	0.7939 (3)	0.6555 (4)	1.2367 (3)	0.0889 (10)	
H13	0.7411	0.6594	1.2922	0.107*	
C14	0.7669 (3)	0.7217 (3)	1.1390 (3)	0.0772 (8)	
H14	0.6977	0.7715	1.1312	0.093*	
N3	0.42879 (17)	0.78365 (18)	0.99087 (16)	0.0513 (5)	
N4	0.20510 (18)	0.8125 (2)	0.96875 (18)	0.0692 (7)	
H4A	0.2161	0.8348	1.0452	0.083*	
H4B	0.1275	0.8098	0.9206	0.083*	
C15	0.3694 (4)	0.5807 (3)	1.1207 (3)	0.0896 (9)	
H15A	0.3615	0.5260	1.1796	0.134*	
H15B	0.4309	0.5519	1.0755	0.134*	
H15C	0.2832	0.5790	1.0667	0.134*	
C16	0.4201 (2)	0.7134 (3)	1.1833 (2)	0.0616 (7)	
C17	0.4444 (3)	0.7462 (4)	1.3083 (3)	0.0825 (9)	
H17	0.4277	0.6830	1.3538	0.099*	
C18	0.4913 (3)	0.8661 (4)	1.3665 (3)	0.0901 (10)	
H18	0.5050	0.8843	1.4500	0.108*	
C19	0.5182 (3)	0.9601 (3)	1.3013 (3)	0.0863 (9)	
H19	0.5513	1.0424	1.3405	0.104*	
C20	0.4963 (2)	0.9325 (3)	1.1778 (2)	0.0642 (7)	
H20	0.5155	0.9965	1.1340	0.077*	
C21	0.44635 (19)	0.8118 (2)	1.1180 (2)	0.0495 (6)	
C22	0.3093 (2)	0.7819 (2)	0.9244 (2)	0.0484 (6)	
C23	0.2832 (2)	0.7442 (2)	0.7916 (2)	0.0515 (6)	
C24	0.1744 (3)	0.7776 (3)	0.7119 (2)	0.0671 (7)	
H24	0.1161	0.8262	0.7416	0.081*	
C25	0.1519 (3)	0.7395 (3)	0.5887 (3)	0.0783 (8)	
H25	0.0783	0.7621	0.5364	0.094*	
C26	0.2365 (3)	0.6692 (3)	0.5435 (3)	0.0816 (9)	
H26	0.2214	0.6442	0.4606	0.098*	
C27	0.3439 (3)	0.6357 (3)	0.6208 (3)	0.0833 (9)	
H27	0.4020	0.5878	0.5900	0.100*	
C28	0.3671 (3)	0.6717 (3)	0.7434 (2)	0.0671 (7)	
H28	0.4402	0.6471	0.7947	0.080*	

### Atomic displacement parameters (Å^2^)

**Table:**

	*U*^11^	*U*^22^	*U*^33^	*U*^12^	*U*^13^	*U*^23^
N1	0.0370 (9)	0.0696 (13)	0.0553 (11)	0.0099 (8)	0.0100 (8)	0.0120 (9)
N2	0.0402 (10)	0.0939 (16)	0.0826 (15)	0.0198 (10)	0.0210 (9)	0.0325 (12)
C1	0.136 (3)	0.0662 (19)	0.087 (2)	−0.0047 (19)	−0.008 (2)	0.0009 (16)
C2	0.0606 (15)	0.0661 (16)	0.0609 (16)	0.0095 (12)	0.0040 (12)	0.0082 (12)
C3	0.097 (2)	0.090 (2)	0.0601 (17)	0.0134 (18)	−0.0004 (15)	0.0146 (15)
C4	0.113 (3)	0.091 (2)	0.085 (2)	0.006 (2)	0.0080 (19)	0.0392 (19)
C5	0.104 (2)	0.0664 (18)	0.102 (3)	−0.0002 (17)	0.011 (2)	0.0254 (18)
C6	0.0624 (15)	0.0669 (17)	0.0694 (17)	0.0030 (12)	0.0100 (12)	0.0065 (13)
C7	0.0348 (10)	0.0583 (14)	0.0591 (14)	0.0068 (9)	0.0100 (9)	0.0097 (11)
C8	0.0341 (10)	0.0521 (12)	0.0541 (13)	0.0056 (9)	0.0086 (9)	0.0012 (10)
C9	0.0395 (11)	0.0524 (13)	0.0521 (13)	0.0010 (9)	0.0099 (9)	0.0036 (10)
C10	0.0629 (15)	0.0667 (15)	0.0732 (17)	0.0202 (12)	0.0252 (12)	0.0186 (13)
C11	0.084 (2)	0.0766 (19)	0.085 (2)	0.0262 (15)	0.0248 (16)	0.0298 (16)
C12	0.081 (2)	0.083 (2)	0.0711 (18)	0.0027 (16)	0.0123 (15)	0.0273 (15)
C13	0.081 (2)	0.129 (3)	0.0676 (18)	0.020 (2)	0.0328 (15)	0.0283 (18)
C14	0.0590 (16)	0.111 (2)	0.0723 (18)	0.0303 (16)	0.0254 (13)	0.0215 (16)
N3	0.0377 (10)	0.0634 (12)	0.0546 (11)	0.0068 (8)	0.0148 (8)	0.0084 (9)
N4	0.0381 (10)	0.1089 (17)	0.0584 (12)	0.0166 (11)	0.0105 (9)	0.0006 (12)
C15	0.102 (2)	0.0692 (19)	0.097 (2)	−0.0074 (16)	0.0294 (18)	0.0172 (16)
C16	0.0501 (13)	0.0753 (17)	0.0631 (16)	0.0099 (12)	0.0184 (11)	0.0142 (13)
C17	0.0803 (19)	0.113 (3)	0.0645 (18)	0.0232 (18)	0.0261 (15)	0.0285 (18)
C18	0.091 (2)	0.119 (3)	0.0580 (17)	0.020 (2)	0.0182 (16)	−0.0026 (19)
C19	0.083 (2)	0.092 (2)	0.074 (2)	0.0097 (17)	0.0135 (16)	−0.0152 (17)
C20	0.0555 (14)	0.0655 (16)	0.0695 (17)	0.0087 (12)	0.0147 (12)	0.0029 (13)
C21	0.0333 (10)	0.0609 (14)	0.0554 (14)	0.0077 (9)	0.0138 (9)	0.0066 (11)
C22	0.0373 (11)	0.0519 (12)	0.0572 (14)	0.0038 (9)	0.0151 (9)	0.0086 (10)
C23	0.0422 (12)	0.0549 (13)	0.0571 (14)	−0.0001 (10)	0.0140 (10)	0.0094 (10)
C24	0.0588 (15)	0.0825 (18)	0.0636 (16)	0.0168 (13)	0.0156 (12)	0.0177 (13)
C25	0.0736 (18)	0.097 (2)	0.0599 (17)	0.0079 (16)	0.0039 (14)	0.0204 (15)
C26	0.083 (2)	0.100 (2)	0.0561 (16)	−0.0026 (17)	0.0186 (15)	0.0018 (15)
C27	0.0714 (18)	0.102 (2)	0.0710 (19)	0.0136 (16)	0.0196 (15)	−0.0133 (16)
C28	0.0532 (14)	0.0801 (17)	0.0639 (16)	0.0121 (12)	0.0113 (12)	−0.0014 (13)

### Geometric parameters (Å, °)

**Table:**

N1—C8	1.295 (3)	N3—C22	1.294 (3)
N1—C7	1.417 (3)	N3—C21	1.421 (3)
N2—C8	1.338 (3)	N4—C22	1.346 (3)
N2—H2A	0.8600	N4—H4A	0.8600
N2—H2B	0.8600	N4—H4B	0.8600
C1—C2	1.490 (4)	C15—C16	1.493 (4)
C1—H1A	0.9600	C15—H15A	0.9600
C1—H1B	0.9600	C15—H15B	0.9600
C1—H1C	0.9600	C15—H15C	0.9600
C2—C3	1.391 (4)	C16—C17	1.394 (4)
C2—C7	1.402 (3)	C16—C21	1.405 (3)
C3—C4	1.332 (4)	C17—C18	1.357 (5)
C3—H3	0.9300	C17—H17	0.9300
C4—C5	1.386 (5)	C18—C19	1.368 (5)
C4—H4	0.9300	C18—H18	0.9300
C5—C6	1.379 (4)	C19—C20	1.375 (4)
C5—H5	0.9300	C19—H19	0.9300
C6—C7	1.369 (3)	C20—C21	1.374 (3)
C6—H6	0.9300	C20—H20	0.9300
C8—C9	1.489 (3)	C22—C23	1.485 (3)
C9—C10	1.377 (3)	C23—C28	1.384 (3)
C9—C14	1.381 (3)	C23—C24	1.388 (3)
C10—C11	1.375 (4)	C24—C25	1.383 (4)
C10—H10	0.9300	C24—H24	0.9300
C11—C12	1.359 (4)	C25—C26	1.359 (4)
C11—H11	0.9300	C25—H25	0.9300
C12—C13	1.347 (4)	C26—C27	1.364 (4)
C12—H12	0.9300	C26—H26	0.9300
C13—C14	1.391 (4)	C27—C28	1.373 (4)
C13—H13	0.9300	C27—H27	0.9300
C14—H14	0.9300	C28—H28	0.9300
			
C8—N1—C7	118.20 (18)	C22—N3—C21	116.93 (17)
C8—N2—H2A	120.0	C22—N4—H4A	120.0
C8—N2—H2B	120.0	C22—N4—H4B	120.0
H2A—N2—H2B	120.0	H4A—N4—H4B	120.0
C2—C1—H1A	109.5	C16—C15—H15A	109.5
C2—C1—H1B	109.5	C16—C15—H15B	109.5
H1A—C1—H1B	109.5	H15A—C15—H15B	109.5
C2—C1—H1C	109.5	C16—C15—H15C	109.5
H1A—C1—H1C	109.5	H15A—C15—H15C	109.5
H1B—C1—H1C	109.5	H15B—C15—H15C	109.5
C3—C2—C7	117.1 (3)	C17—C16—C21	116.7 (3)
C3—C2—C1	121.9 (3)	C17—C16—C15	122.3 (3)
C7—C2—C1	121.0 (3)	C21—C16—C15	121.1 (2)
C4—C3—C2	123.0 (3)	C18—C17—C16	122.9 (3)
C4—C3—H3	118.5	C18—C17—H17	118.5
C2—C3—H3	118.5	C16—C17—H17	118.5
C3—C4—C5	120.2 (3)	C17—C18—C19	119.4 (3)
C3—C4—H4	119.9	C17—C18—H18	120.3
C5—C4—H4	119.9	C19—C18—H18	120.3
C6—C5—C4	118.3 (3)	C18—C19—C20	119.8 (3)
C6—C5—H5	120.9	C18—C19—H19	120.1
C4—C5—H5	120.9	C20—C19—H19	120.1
C7—C6—C5	121.8 (3)	C21—C20—C19	121.0 (3)
C7—C6—H6	119.1	C21—C20—H20	119.5
C5—C6—H6	119.1	C19—C20—H20	119.5
C6—C7—C2	119.5 (2)	C20—C21—C16	120.1 (2)
C6—C7—N1	120.1 (2)	C20—C21—N3	120.4 (2)
C2—C7—N1	120.2 (2)	C16—C21—N3	119.4 (2)
N1—C8—N2	123.3 (2)	N3—C22—N4	123.7 (2)
N1—C8—C9	118.86 (18)	N3—C22—C23	119.19 (19)
N2—C8—C9	117.84 (19)	N4—C22—C23	117.14 (19)
C10—C9—C14	117.2 (2)	C28—C23—C24	117.7 (2)
C10—C9—C8	120.7 (2)	C28—C23—C22	120.2 (2)
C14—C9—C8	122.1 (2)	C24—C23—C22	122.1 (2)
C11—C10—C9	121.2 (3)	C25—C24—C23	120.7 (3)
C11—C10—H10	119.4	C25—C24—H24	119.7
C9—C10—H10	119.4	C23—C24—H24	119.7
C12—C11—C10	120.8 (3)	C26—C25—C24	120.5 (3)
C12—C11—H11	119.6	C26—C25—H25	119.8
C10—C11—H11	119.6	C24—C25—H25	119.8
C13—C12—C11	119.5 (3)	C25—C26—C27	119.5 (3)
C13—C12—H12	120.3	C25—C26—H26	120.2
C11—C12—H12	120.3	C27—C26—H26	120.2
C12—C13—C14	120.5 (3)	C26—C27—C28	120.8 (3)
C12—C13—H13	119.8	C26—C27—H27	119.6
C14—C13—H13	119.8	C28—C27—H27	119.6
C9—C14—C13	120.9 (3)	C27—C28—C23	120.9 (3)
C9—C14—H14	119.6	C27—C28—H28	119.6
C13—C14—H14	119.6	C23—C28—H28	119.6
			
C7—C2—C3—C4	−1.5 (5)	C21—C16—C17—C18	−0.1 (4)
C1—C2—C3—C4	179.6 (3)	C15—C16—C17—C18	179.6 (3)
C2—C3—C4—C5	1.5 (6)	C16—C17—C18—C19	−0.9 (5)
C3—C4—C5—C6	−0.6 (6)	C17—C18—C19—C20	0.7 (5)
C4—C5—C6—C7	−0.2 (5)	C18—C19—C20—C21	0.6 (4)
C5—C6—C7—C2	0.2 (4)	C19—C20—C21—C16	−1.6 (3)
C5—C6—C7—N1	175.4 (3)	C19—C20—C21—N3	−177.7 (2)
C3—C2—C7—C6	0.6 (4)	C17—C16—C21—C20	1.4 (3)
C1—C2—C7—C6	179.5 (3)	C15—C16—C21—C20	−178.3 (3)
C3—C2—C7—N1	−174.6 (2)	C17—C16—C21—N3	177.5 (2)
C1—C2—C7—N1	4.3 (4)	C15—C16—C21—N3	−2.2 (3)
C8—N1—C7—C6	95.6 (3)	C22—N3—C21—C20	−98.8 (2)
C8—N1—C7—C2	−89.2 (3)	C22—N3—C21—C16	85.1 (3)
C7—N1—C8—N2	0.4 (3)	C21—N3—C22—N4	4.2 (3)
C7—N1—C8—C9	179.91 (19)	C21—N3—C22—C23	−175.76 (19)
N1—C8—C9—C10	−18.2 (3)	N3—C22—C23—C28	22.1 (3)
N2—C8—C9—C10	161.3 (2)	N4—C22—C23—C28	−157.9 (2)
N1—C8—C9—C14	161.7 (2)	N3—C22—C23—C24	−159.0 (2)
N2—C8—C9—C14	−18.8 (3)	N4—C22—C23—C24	21.1 (3)
C14—C9—C10—C11	0.3 (4)	C28—C23—C24—C25	−0.1 (4)
C8—C9—C10—C11	−179.8 (2)	C22—C23—C24—C25	−179.1 (2)
C9—C10—C11—C12	0.6 (5)	C23—C24—C25—C26	−0.4 (4)
C10—C11—C12—C13	−0.3 (5)	C24—C25—C26—C27	0.4 (5)
C11—C12—C13—C14	−1.0 (5)	C25—C26—C27—C28	0.2 (5)
C10—C9—C14—C13	−1.5 (4)	C26—C27—C28—C23	−0.7 (5)
C8—C9—C14—C13	178.5 (3)	C24—C23—C28—C27	0.7 (4)
C12—C13—C14—C9	1.9 (5)	C22—C23—C28—C27	179.7 (3)

### Hydrogen-bond geometry (Å, °)

**Table:**

*D*—H···*A*	*D*—H	H···*A*	*D*···*A*	*D*—H···*A*
N2—H2B···N3	0.86	2.25	3.049 (3)	156
N4—H4B···N1^i^	0.86	2.24	3.016 (3)	151

Symmetry codes: (i) *x*−1, *y*, *z*.
